# Red Clover (*Trifolium pratense*) and Zigzag Clover (*T. medium*) – A Picture of Genomic Similarities and Differences

**DOI:** 10.3389/fpls.2018.00724

**Published:** 2018-06-05

**Authors:** Jana Dluhošová, Jan Ištvánek, Jan Nedělník, Jana Řepková

**Affiliations:** ^1^Department of Experimental Biology, Faculty of Science, Masaryk University, Brno, Czechia; ^2^Agricultural Research, Ltd., Troubsko, Czechia

**Keywords:** zigzag clover karyotype, sequencing, FISH, comparative analysis, centromeric repeats

## Abstract

The genus clover (*Trifolium* sp.) is one of the most economically important genera in the Fabaceae family. More than 10 species are grown as manure plants or forage legumes. Red clover’s (*T. pratense*) genome size is one of the smallest in the *Trifolium* genus, while many clovers with potential breeding value have much larger genomes. Zigzag clover (*T. medium*) is closely related to the sequenced red clover; however, its genome is approximately 7.5x larger. Currently, almost nothing is known about the architecture of this large genome and differences between these two clover species. We sequenced the *T. medium* genome (2n = 8x = 64) with ∼23× coverage and managed to partially assemble 492.7 Mbp of its genomic sequence. A thorough comparison between red clover and zigzag clover sequencing reads resulted in the successful validation of 7 *T. pratense*- and 45 *T. medium*-specific repetitive elements. The newly discovered repeats led to the set-up of the first partial *T. medium* karyotype. Newly discovered red clover and zigzag clover tandem repeats were summarized. The structure of centromere-specific satellite repeat resembling that of *T*. *repens* was inferred in *T. pratense*. Two repeats, TrM300 and TrM378, showed a specific localization into centromeres of a half of all zigzag clover chromosomes; TrM300 on eight chromosomes and TrM378 on 24 chromosomes. A comparison with the red clover draft sequence was also used to mine more than 105,000 simple sequence repeats (SSRs) and 1,170,000 single nucleotide variants (SNVs). The presented data obtained from the sequencing of zigzag clover represent the first glimpse on the genomic sequence of this species. Centromeric repeats indicated its allopolyploid origin and naturally occurring homogenization of the centromeric repeat motif was somehow prevented. Using various repeats, highly uniform 64 chromosomes were separated into eight types of chromosomes. Zigzag clover genome underwent substantial chromosome rearrangements and cannot be counted as a true octoploid. The resulting data, especially the large number of predicted SSRs and SNVs, may have great potential for further research of the legume family and for rapid advancements in clover breeding.

## Introduction

The family Fabaceae is one of the largest and the most economically important families of flowering plants. The genus clover (*Trifolium* sp.) comprises of approximately 250 species, 20 of which have been commercially cultivated, making it one of the largest genera in this family (Ellison et al., 2006). Similar to other leguminous species, it is capable of fixing atmospheric nitrogen, which results in high protein forage as well as a reduced need for nitrogen fertilizer input (Taylor and Quesenberry, 1996). These beneficial attributes have determined its use as a manure plant or forage legume in livestock farming systems.

Red clover (*Trifolium pratense* L.) is a high-quality fodder crop that is widely cultivated in most temperate regions both within Europe and worldwide. It is sown as a companion crop and a green manure crop to increase soil fertility. The main disadvantage of its breeding is a low persistency which is a highly complex trait that cannot be easily modified even with utilization of modern methods based on genetic improvement (Řepková and Nedělník, 2014). Introduction of appropriate trait from closely related zigzag clover (*Trifolium medium* L.) by means of artificial interspecific hybridization has been performed and led to a viable hybrid progeny *T*. *pratense* × *T*. *medium* (Řepková et al., 1991, 2006b). Hybrids were thoroughly inspected on the levels of morphological, agronomic and reproductive traits and feeding characteristics (Jakešová et al., 2011, 2014) and plants exceeded high quality fodder of red clover. Recently, subsequent hybrid generations were further evaluated from the viewpoint of genetic impact on variability in chromosome number and rDNA loci at the level of individual plants (Dluhošová et al., 2016). Hybrid plants demonstrated extraordinary variability within chromosome counts, high variability was also observed within number and arrangement of 5S and 45S rDNA loci with unique or novel rDNA loci pattern. However, thorough input information about both parental genomes with the knowledge of similarities and differences between them is still missing which prevents us from precise identification of introgressed features on the level of individual hybrid plants.

As for the available genomic data of the red clover, the tetraploid variety Tatra (Ištvánek et al., 2014) and diploid variety Milvus B (De Vega et al., 2015) have been recently *de novo* sequenced, the resulting genome assemblies were precisely annotated and both the repetitive and coding proportion of the genome were described in detail, which provides us with input sequencing data for desired comparative analysis. However, to our best knowledge, almost no information regarding the complex polyploid genome and respective sequencing data are available for the wild zigzag clover. Comparative analysis of these two species has thus not yet been possible, even though the available basic genomic characteristics of both species indicate potential major differences which are yet to be revealed. In spite of the close phylogenetic relatedness of both clovers belonging to the distinct clade within the subgenus *Trifolium* (Watson et al., 2000; Ellison et al., 2006; Vižintin et al., 2006), they manifest some striking differences such as different basic chromosome number (x = 7 in red clover and x = 8 in zigzag clover) or substantially different genome size. Zigzag clover genome of 3,154 Mbp (1C = 3.23 pg) is approximately 7.5× larger than the red clover genome of 418 Mbp (1C = 0.43 pg) (Vižintin et al., 2006). Presented features imply major genomic rearrangements as well as reconstitution and potential expansions of repetitive elements that took place during the red clover and zigzag clover speciation within the *Trifolium* subgenus. Knowledge about the repetitive content, especially individual species-specific tandem and interspersed repeats, can create basis for precise hybrid state assessment, as was shown, e.g., in well-known cereal hybrids *Hordeum chilense* × *Secale africanum* (Schwarzacher et al., 1989) or *Festulolium* (Kopecký et al., 2006). Thorough analysis based on the sequencing data is thus emerging as essential for future identification of preserved post-hybridization genomic changes.

In this paper we present the results obtained from the comparative analysis between red clover and zigzag clover based on the Illumina sequencing of the zigzag clover genome with the coverage of approximately 23×. The comparison is aimed mainly at the repeat content characterization focused on discovering and verification of species-specific repetitive elements using fluorescent *in situ* hybridization (FISH). Nevertheless, the obtained sequencing data were also used for prediction of potential DNA markers. All our presented results thus create a complex picture of genomic similarities and differences that can set the basis not only for the future detailed analysis of the hybrid progeny, but also for the practical utilization of wild zigzag clover in the forthcoming breeding programs.

## Materials and Methods

### Plant Material

Plants of octoploid (2n = 8x = 64) zigzag clover (*T*. *medium*) clone 10/8 were obtained from the breeding facility of Dr. Hana Jakešová, Clovers and Grass Plant Breeding (Hladké Životice, Czechia). Leaves were collected from 30-day-old, greenhouse-grown plants. Genomic DNA for sequencing was extracted from nuclei isolated from ∼10 g of young leaves from 16 cloned plants using the method described by Zhang et al. (1995). Genomic DNA for purposes other than sequencing was extracted from leaves as described by Dellaporta et al. (1983).

### Illumina Sequencing

Zigzag clover paired-end genomic DNA library was constructed by IGA Technology Services (Udine, Italy) using a TruSeq DNA-seq kit. Clusters were generated in a flow cell by the cBot system (IGA Technology Services S.R.L., Udine, Italy), and the library was sequenced on a HiSeq 2000 using a standard Illumina sequencing workflow. The resulting 100-nucleotide-long paired-end reads were obtained from a single genomic library with an insert size of 300–1200 bp. A total number of 724.4 million raw reads were evaluated by FastQC^1^, and relics of sequencing adapters and low-quality bases were discarded using the FASTX-toolkit^2^. Sequence reads are available at the Sequence Read Archive of NCBI under accession SRP071842, and the project has been deposited in the DDBJ/EMBL/GenBank under Accession No. LXQA00000000. The version described in this paper is version LXQA00000000. The zigzag clover draft genomic sequence was created the same way as described by Ištvánek et al. (2014). Adapter sequence and low-quality reads were removed using the Echo v1.11 (Kao et al., 2011) program, *de novo* assembly was performed using the Abyss assembler v1.3.3 (Simpson et al., 2009).

### Repeat Content Characterization

Sequencing reads were used for the repeat content characterization of the zigzag clover genome both independently and in direct comparison with red clover by means of comparative clustering. The sequencing reads from red clover used in this comparative approach were obtained from previous studies (Ištvánek et al., 2014). Repeat content characterizations of both individual and comparative approaches were carried out by an all-to-all similarity comparison and by graph-based clustering using RepeatExplorer (Novák et al., 2013), a clustering-based repeat identification pipeline implemented in the Galaxy platform^3^.

A total of 4,022,796 (∼0.1×) Illumina reads were used as input for individual zigzag clover repeat content characterization. Repetitive sequences were sorted using a similarity-based clustering analysis, while groups of reads (clusters) containing more than 0.1% of used reads were inspected more closely. The annotation of resulting clusters was based on results from several analyses: graphical representations of all clusters were examined in SeqGrapheR (Novák et al., 2010) in order to identify tandem repeats. Structural features were identified using Dotter (Sonnhammer and Durbin, 1995). The identification of insertion sites in potential transposable elements was performed by program clview^4^. Additionally, similarity hits to known repeats included in various databases, such as RepeatMasker, with Repbase (implemented in RepeatExplorer) (Jurka et al., 2005) and BLAST (Altschul et al., 1990) searches of contigs assembled by clusters with CAP3 (Huang and Madan, 1999) were taken into account.

The repeat content of zigzag clover was directly compared to that of red clover by means of comparative clustering. Because of different ploidy levels and genome sizes, it was necessary to properly choose the number of reads that would be used for repeat content analysis. The genome content of both plants was measured by flow cytometry [Partec Ploidy Analyser-I (PA-I), Germany]. The internal reference standards used to measure red clover and zigzag clover were *Glycine max* and *Zea mays*, respectively. Only partial, equal proportions of sequences corrected for genome size and ploidy level were randomly chosen using a custom R script. The resulting pooled set of 127,504,257 bp from red clover and 208,446,121 bp from zigzag clover was used as an input for clustering in RepeatExplorer. The annotation of the resulting clusters was performed as described above. Each cluster was considered species-specific if the proportion of the other species in the whole cluster or selected contigs was less than 1%. The clusters evaluated as tandem repeats were analyzed by Tandem Repeats Finder (Benson, 1999) in order to discover their consensus monomer. Other species-specific clusters were analyzed in detail using SeqGrapheR (Novák et al., 2010) to identify the most conserved parts of their contigs suitable for the design of FISH probes. All of the analyzed FISH probes were subjected to pairwise hybridization with each other on both red clover and zigzag clover chromosomes.

### Probe Design and Production

Fluorescent *in situ* hybridization probes for tandem repeats with a short consensus monomer (up to 80 bp) were synthesized as oligonucleotides by Sigma-Aldrich (Haverhill, United Kingdom). Unmodified lyophilized DNA oligonucleotides corresponding to both complementary DNA strands were resuspended in water to a final concentration of 100 μM. Equal volumes of both oligonucleotides were mixed together in a tube and heated to 95°C for 5 min. Immediately after heating, the tube was transferred to a beaker containing 0.5 L of ∼95°C water. After slow cooling at room temperature to ∼30°C, the resulting double-strand DNA was quantified using a NanoDrop spectrophotometer (Thermo Scientific, Vienna, Austria). FISH probes from sequences other than short tandem repeats were designed for the most conserved part of their contigs. Probe sequences were selected manually to obtain a high level of sequence complexity with sufficient length and coverage. A specific pair of primers was selected for each element using Primer3 (Untergasser et al., 2012), OligoCalc (Kibbe, 2007), OligoAnalyzer v3.1 (Owczarzy et al., 2008), and PrimerBlast (Ye et al., 2012). Probe sequences were amplified by PCR containing 1× GoTaq Reaction buffer (Promega), 0.2 mM dNTPs, 1 μM primers, 0.5 U of Taq Polymerase (Promega) and 20 ng of gDNA. PCR products were separated by agarose electrophoresis, excised from the gel, purified with a PCR purification kit (Qiagen) and quantified using a NanoDrop Spectrophotometer.

### Probe Labeling and FISH

Root tips from red clover and zigzag clover were synchronized overnight on ice and stored in Carnoy’s fixative at -20°C. Chromosome spreads were prepared after pretreatment with pectolytic enzyme mixture (0.3% pectolyase, 0.3% cellulase, and 0.3% cytohelicase in 1× citrate buffer) by the SteamDrop method according to Kirov et al. (2014) with a Double SteamDrop modification. All of the probes were labeled by nick translation using Biotin or DIG Nick Translation Mix (Roche). Then, 100 ng of labeled probe was ethanol precipitated and resuspended in 25 μl of hybridization buffer containing 50% formamide and 10% dextran sulfate in 2× SSC. The mixture was denatured by incubation at 95°C for 5 min and immediately placed on ice. Slides with chromosome spreads were treated with 100 μg/ml RNase A (Sigma) in 2× SSC for 1 h at 37°C, washed twice for 5 min in 2× SSC, treated with 0.1 mg/ml pepsin in 10 mM HCl for 2 min at 37°C, washed as before, post-fixed in 4% formaldehyde in 2× SSC, washed again and dehydrated in an increasing ethanol series (70, 90, and 96% ethanol, 5 min each). The probes were applied to suitable chromosome spreads, codenaturated at 80°C for 2 min and left to hybridize overnight at 37°C in a humid box. Post-hybridization washing was carried out at 42°C with the following steps: 2× SSC twice for 5 min, 10% formamide/0.1× SSC twice for 5 min, 2× SSC for 5 min and 4× SSC/0.05% Tween-20. Biotin- or DIG-labeled probes were immunodetected with streptavidin-Cy3 (GE Healthcare, Buckinghamshire, United Kingdom; dilution 1:1000) and anti-DIG-FITC (Roche, Mannheim, Germany; dilution 1:200) antibodies. The slides were counterstained with DAPI in Vectashield (Vector Laboratories, Burlingame, CA, United States). An Olympus BX-51 fluorescence microscope was used for sample evaluation; the micrographs were captured using an Olympus DP72 CCD camera and Cell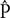 imaging system (Olympus). Suitable images were pseudocolored and merged in Adobe CS6 Photoshop.

### DNA Markers

Simple sequence repeat (SSR) loci within the partially assembled genomic sequence of zigzag clover were identified by SSR Locator (da Maia et al., 2008). Each SSR site was defined as a monomer occurring at least 12×, a dimer at least 6×, tri- and tetramers at least 4×, and penta- and hexamers at least 3×. Primers with T_m_ near 60°C were designed for potential SSR markers, and the number of PCR products was predicted for each primer pair.

To identify potential single nucleotide variants (SNVs) in zigzag clover, the reference sequence of red clover (Ištvánek et al., 2014) was used. Zigzag clover sequencing reads were mapped to the reference using bwa v0.7.5 (Li and Durbin, 2010). SAMTools v0.1.19 (Li et al., 2009) was used to convert between BAM and SAM formats; the sorting of mapped reads, marking PCR duplicates, and indexing were performed by Picard v1.80^5^. To remap sequence reads in proximity to InDel, the recalibration of base qualities and SNV calling GATK v2.7 (McKenna et al., 2010) was performed. Custom Perl scripts were used to further process and identify species-specific and interspecific markers.

## Results

### Genome Assembly

The Illumina sequencing of zigzag clover resulted in 724.4 million 100-bp-long paired-end reads from a single genomic library. The average fragment size of the genomic library was 750 bp, and raw genome coverage of ∼23× was achieved. Raw data were filtered as described above, leaving an average genome coverage of 21.1×. Features of this partially assembled, 492.7 Mbp-long genomic sequence are described in Supplementary Table S1.

### Repeat Content Characterization

**FIGURE 1:**
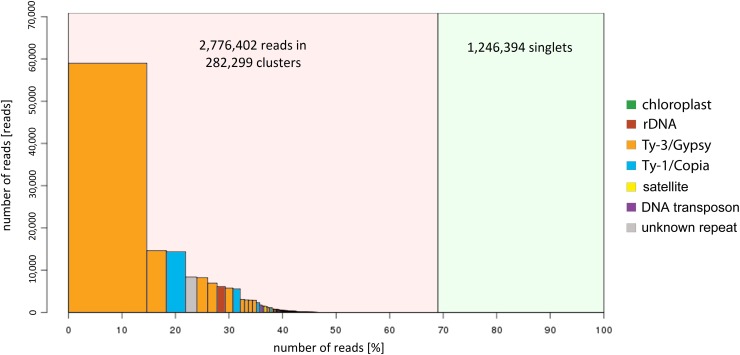
Size distribution and repeat composition of clusters generated by the similarity-based partitioning of *Trifolium medium* reads. The cumulative proportion of clusters in the genome is shown along the *x*-axis. Bars on the histogram represent individual clusters; bar height corresponds to the number of reads in the clusters and color to the types of repetitive element.

A total of 4,022,796 sequencing reads of zigzag clover were used to predict the proportion of repetitive elements in the newly sequenced genome. In the clustering-based approach of the RepeatExplorer pipeline, the clusters contained 69% of all analyzed reads, with 32% being assigned to the nine largest clusters representing the most abundant repetitive elements in the genome (**Figure 1**). A total of 14% of the analyzed reads belonged to the largest cluster, representing elements from the lineage of Chromoviruses from Ty3/Gypsy retrotransposons. The lineages of Ty3/Gypsy retrotransposons occupy as much as 28.14% of the genome, making retrotransposons the most abundant class of repetitive elements. Together with Ty1/Copia elements, they form more than one-third (36.66%) of the zigzag clover genome (Supplementary Table S2). In both cases, all of the main retrotransposon lineages are present in the genome of zigzag clover, although their abundances differ substantially. The present DNA transposons (2.89%) belong to all main groups, with PIF/Harbinger and Mutator forming 57.4% of all DNA transposons found. In total, detailed inspection and annotation successfully described 46.67% of the genome size consisting of different repetitive elements.

In addition, a direct comparison of the repeat content of both the zigzag clover and red clover genomes was performed by comparative clustering. The genome content (2C) estimated by flow cytometry was 1.963 pg (*SD*: 0.029) for red clover and 7.054 pg (*SD*: 0.054) for zigzag clover. According to the octoploid nature of the zigzag clover genome, only half of the DNA content was considered as if both plants had equal ploidy levels, so that the coverage of the haploid genome was the same. The measured values were converted to Mbp according to Dolezel et al. (2003). For the purposes of comparative clustering, the genome sizes of tetraploid red clover and tetraploid zigzag clover were calculated as 810 and 1,457 Mbp. A total of 1,307,142 reads from red clover and 2,347,960 reads from zigzag clover were pooled together and subjected to repeat content characterization.

The similarity-based clustering of the reads resulted in 286,417 clusters containing from 2 to 37,866 reads. The clusters included 65.5% of all analyzed reads; the remaining 1,255,666 reads were classified as singlets. The proportions of reads included in the resulting clusters from red clover and zigzag clover were 61.2 and 67.9%, respectively. A total of 336 largest clusters containing at least 0.01% of all analyzed reads represented 41.6% of all analyzed reads, and 286,081 smaller clusters with 2–363 reads contained a total of 870,253 reads, which was 23.9% of the input.

**FIGURE 2:**
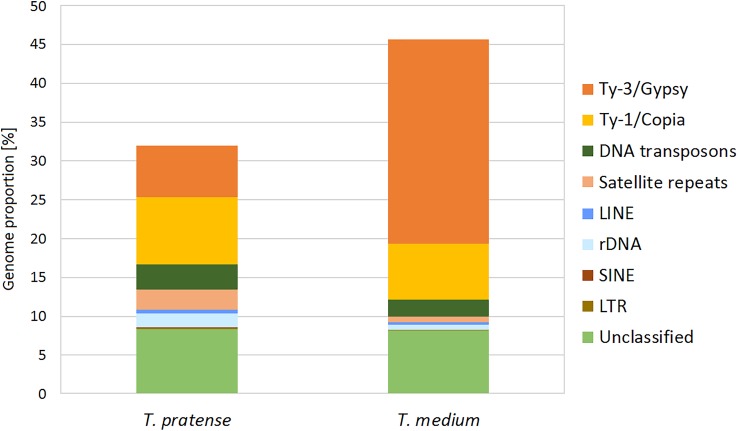
Repeat composition in red clover and zigzag clover estimated from Illumina sequencing data visualized in the graph.

The further inspection of the 336 largest clusters, such as an evaluation of the presence of insertion sites or subrepeats, resulted in the successful classification of repeat types in the majority of these clusters. A summary of the classification and the genome proportion of each repeat type in both species are shown in **Figure 2** and **Table 1**.

**Table 1:** Repeat composition of the red clover and zigzag clover genomes estimated from Illumina sequencing data by comparative clustering.

Classification			Genome proportion (%)	
Repeat type	Family	Lineage	*T*. *pratense*	*T*. *medium*
Retroelements			15.93	33.81
	Ty3/Gypsy		6.65	26.29
		Chromovirus	3.27	17.03
		Athila	1.95	6.04
		Tat/Ogre	1.17	2.46
		other	0.27	0.76
	Ty1/Copia		8.59	7.16
		Maximus/SIRE	4.67	4.30
		Angela	0.95	0.37
		Ivana/Oryco	0.36	0.81
		Tork	0.40	0.67
		AleII	0.47	0.24
		Bianca	0.44	0.16
		TAR	0.30	0.09
		AleI/Retrofit	0.06	0.02
		Other	0.94	0.50
	LINE		0.45	0.28
	SINE		0.10	0.02
	Other		0.14	0.07
DNA transposons			3.22	2.19
	Mutator		1.05	0.78
	Mariner		0.57	0.32
	RC/Helitron		0.53	0.19
	hAT		0.36	0.32
	PIF/Harbinger		0.50	0.12
	CACTA		0.12	0.43
	Other		0.09	0.03
Satellite repeats			2.63	0.82
rDNA			1.79	0.59
Unclassified			8.37	8.24
Total			31.93	45.65

Although the most prevalent repetitive elements in both species belong to LTR retroelements, zigzag clover has a much larger proportion of Ty3/Gypsy retroelements. This difference in the proportion of Ty3/Gypsy, especially the lineage chromovirus, seemed to be the main cause of the different proportion of the whole repetitive fraction. Other types of repetitive elements did not show such substantial differences; their proportions in both species were more or less the same.

A detailed analysis was performed for species-specific clusters in which the proportion of the other species was less than 1% of all of the containing reads. A total of 7 and 45 species-specific clusters were identified for red clover and zigzag clover, respectively (**Table 2**). A subset of 6 and 18 specific clusters was chosen for validation based on the length of the assembled contigs and their coverage (**Table 3**). FISH probes were designed from one to several merged contigs depending on their total length and coverage.

**Table 2:** Number of species-specific clusters according to their classification.

	Ty3/Gypsy	Ty1/Copia	Tandem repeat	DNA transposon	Unknown	Σ
*T. pratense*		1	3		3	7
*T. medium*	5	5	2	1	32	45

**Table 3:** Selected *T*. *pratense*- and *T*. *medium*-specific contigs, number of comprising reads and their annotation.

	Number of *T. pratense* reads	Number of *T. medium* reads	Σ	% of the other species in selected contigs^∗^	Cluster annotation
***T. pratense***					
CL12	19,305	4	19,309	0.02	Tandem repeat 38 bp
CL55	8,440	404	8,844	0.46	Unknown
CL127	3,171	26	3,197	0	Unknown
CL167	2,198	10	2,208	0	Tandem repeat 1,586 bp
CL172	2,031	19	2,050	0.62	Unknown
CL198	1,297	226	1,523	0.34	Tandem repeat 29 bp
***T. medium***					
CL9	9	21,219	21,228	0	Unknown
CL17	1	16,378	16,379	0	Unknown
CL50	949	8,494	9,443	0	Ty3/Gypsy Tat/Ogre integrase
CL53	3,410	5,600	9,010	0	Ty1/Copia Maximus/SIRE GAG
CL64	53	7,798	7,851	0	Unknown
CL102	9	4,758	4,767	0	Tandem repeat 179 bp
CL106	0	4,392	4,392	0	Unknown
CL110	20	4,172	4,192	0	Ty1/Copia
CL122	6	3,368	3,374	0.01	Ty1/Copia Maximus/SIRE GAG
CL140	0	2,997	2,997	0	Unknown
CL146	239	2,518	2,757	0	Unknown
CL150	3	2,622	2,625	0	Unknown
CL153	0	2,566	2,566	0	Unknown
CL164	0	2,266	2,266	0	Unknown
CL195	0	1,603	1,603	0	Ty1/Copia
CL196	1	1,600	1,601	0	Unknown
CL197	0	1,570	1,570	0	Unknown
CL354	63	252	315	0.80	Tandem repeat 60 bp

^∗^*Contigs used for design of FISH probes*.

### FISH Validation

Fluorescent *in situ* hybridization probes for selected tandem repeats with a short monomer sequence (CL12, CL198, and CL354) were synthesized as complementary oligonucleotides with a length of up to 80 bp containing one to several monomer motifs. A consensus monomer sequence identified for all species-specific tandem repeat clusters is listed in Supplementary Table S3. FISH probes for other species-specific clusters were prepared from amplified DNA resulting from PCR reactions with cluster-specific primers (Supplementary Table S4). These PCR reactions were also used as a preliminary validation of the species-specificity and of the predicted length. The products of amplification from all of the studied clusters were present in the expected species alone; their lengths exactly matched the predicted ones in all cases (Supplementary Figure S1).

**FIGURE 3:**
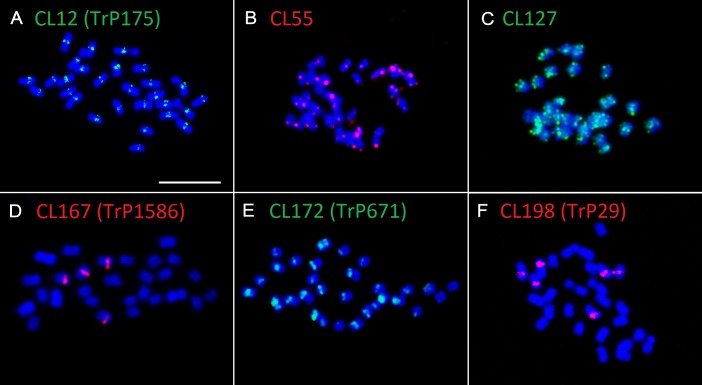
Hybridization patterns of six *Trifolium pratense*-specific repeats. **(A)** Probes derived from CL12 (tandem repeat TrP175) show centromeric localization on all red clover chromosomes. Dispersed fluorescent patterns present in CL55 **(B)** and CL127 **(C)** hybridization. Probes derived from CL167 (tandem repeat TrP1586) **(D)**, CL172 (tandem repeat TrP671) **(E)**, and CL198 (tandem repeat TrP29) **(F)** hybridized to the pericentromeric region on 4, 28, and 6 chromosomes, respectively. Chromosomes of red clover are counterstained with DAPI. Fluorescent probes are labeled with digoxigenin visualized by antiDIG-FITC antibodies (green) or biotin visualized by streptavidin-Cy3 antibodies (red). Scale bar: 10 μm.

The validation of species-specificity was also performed by FISH on both red clover and zigzag clover chromosome spreads. All of the analyzed elements hybridized only to chromosomes of the predicted species; no fluorescent signal was observed in the other species. Four studied elements specific to red clover hybridized to well-distinguishable positions on several chromosomes (**Table 4**). Probes derived from CL12 and CL172 hybridized to the centromeric position of all 28 chromosomes. We presume that these elements might be directly connected to the centromere constitution as centromere-specific repeats. Probes from CL167 and CL198 hybridized to the pericentromeric region on 4 and 6 chromosomes, respectively. Probes derived from CL55 and CL127 showed a uniformly dispersed fluorescent signal along all red clover chromosomes. The fluorescent signals of analyzed elements are shown in **Figure 3**.

**Table 4:** Table of all of the newly discovered *Trifolium*-specific tandem repeats.

Name	Cluster	Proportion (%)	Basic motif (bp)	Annotation	Localization
***T. pratense***					
TrP175	CL12	1.48	175	Centromeric repeat	All chromosomes
TrP1586	CL167	0.169	1,586	Pericentromeric repeat	4/28 chromosomes
TrP671	CL172	0.16	671^∗^	Pericentromeric repeat	All chromosomes
TrP29	CL198	0.10	29	Pericentromeric repeat	6/28 chromosomes
***T. medium***					
TrM378	CL9	0.906	378^∗^	Centromeric repeat	32/64 chromosomes
TrM300	CL17	0.70	300^∗^	Centromeric repeat	32/64 chromosomes
TrM179	CL102	0.20	179	Subtelomeric repeat	24/64 chromosomes
TrM60	CL354	0.01	60	Pericentromeric repeat	4/64 chromosomes

^∗^*Precise length of basic motif cannot be determined, the presented value is the length of PCR-amplified segment*.

**FIGURE 4:**
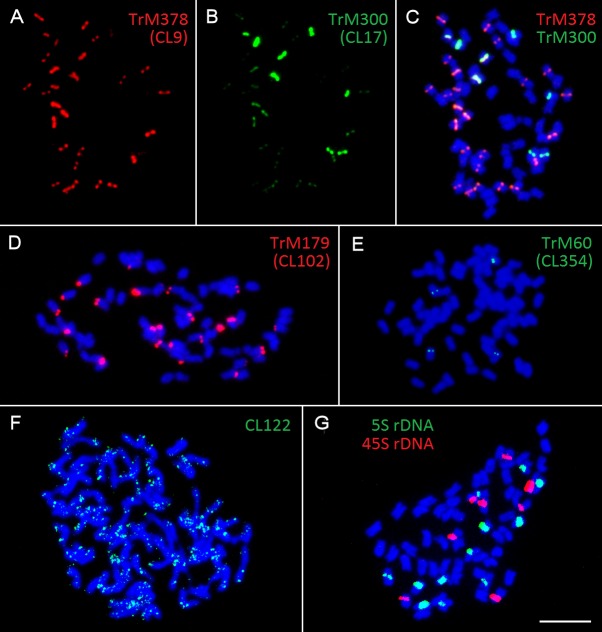
Patterns of fluorescent signals from several different FISH probes on *T*. *medium* chromosomes. Fluorescent signals of elements TrM378 and TrM300 derived from CL9 and CL17 and their localization on zigzag clover chromosomes. Both TrM378 probes **(A)** and TrM300 probes **(B)** hybridize to the same positions on 32 chromosomes of zigzag clover. Merged picture **(C)** shows the different overall hybridization signal on each chromosome, with eight chromosomes having a higher proportion of TrM300 (green) and 24 chromosomes having a higher proportion of TrM378 (red). **(D)** Probes of TrM179 derived from CL102 hybridize as a satellite to the terminal part of the short arm of 24 chromosomes. **(E)** Probes of TrM60 derived from CL354 hybridize to the pericentromeric region of four chromosomes. **(F)** Dispersed character of hybridization, which was observed in 18 repetitive elements shown on exemplary CL122. **(G)** Hybridization pattern of 5S (green) and 45S (red) rDNA FISH probes (Dluhošová et al., 2016). All of the chromosomes are counterstained with DAPI. Fluorescent probes of TrM300 **(B)**, TrM60 **(E)**, CL122 **(F)**, and 5S rDNA **(G)** are labeled with digoxigenin visualized by antiDIG-FITC antibodies (green); probes of TrM378 **(A)**, TrM179 **(D)**, and 45S rDNA **(G)** are labeled with biotin visualized by streptavidin-Cy3 antibodies (red). Scale bar: 10 μm.

Fluorescent *in situ* hybridization was also performed for all repetitive elements specific to zigzag clover. Only four elements hybridized to well-distinguishable positions on several chromosomes (**Figures 4A–E**); the remaining (18 elements) hybridized dispersedly along all of the chromosomes of zigzag clover without any specific pattern (**Figure 4F**). The probes derived from CL9 and CL17 hybridized to the centromeric position of 32 chromosomes. Both probes hybridized to the same chromosomes with the same localization, although the proportion of each element differed on individual chromosomes (**Figures 4A–C**). Eight chromosomes showed a higher proportion of CL17 elements; the remaining 24 chromosomes had a higher proportion of elements from CL9.

The probes derived from CL102 hybridized as a satellite on the terminal part of the short arm of 24 chromosomes of zigzag clover. The probes derived from CL354 hybridized to the pericentromeric region of four chromosomes. The localization of both CL102 and CL354 fluorescent signals is shown in **Figures 4D,E**.

**FIGURE 5:**
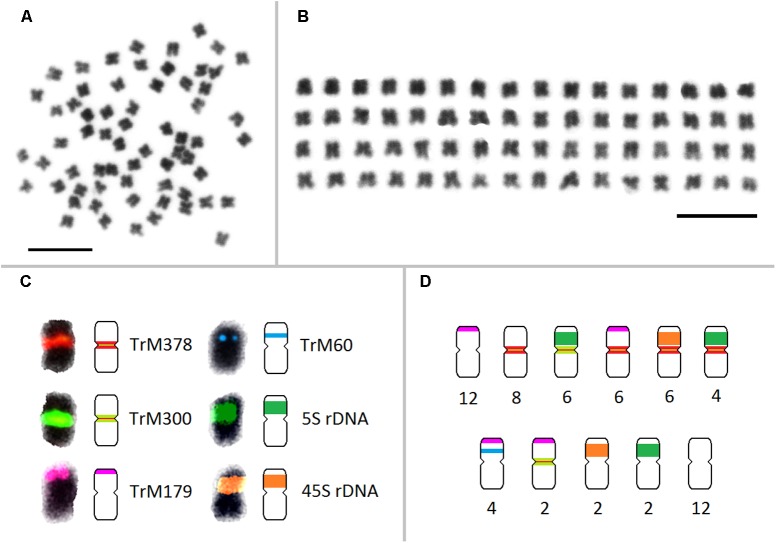
Karyotype and a simplified graphical representation of all 64 chromosomes of zigzag clover showing the localization of repetitive elements TrM378, TrM300, TrM179, TrM60, and 5S and 45S rDNA loci. **(A)** Metaphase chromosomes of zigzag clover. **(B)** Metaphase zigzag clover chromosomes arranged into the karyotype. Chromosomes were not put into pairs because of insufficient differences among individual chromosomes. **(C)** Localization of fluorescent signals of individual tandem repeats with their corresponding graphical representations. Hybridization signals of TrM378 and TrM300 are summarized within one signal but represented by two different schemes considering the predominant element. **(D)** Counts of the individual type of chromosomes presented in all 64 chromosomes of zigzag clover. Scale bar: 10 μm.

All zigzag clover-specific probes were subjected to pair-wise hybridization with each other. The results were also merged with previously published 5S and 45S rDNA hybridization (Dluhošová et al., 2016; **Figure 4G**) to further assign analyzed elements to individual chromosomes. A simplified graphical representation showing the localization of CL9, CL17, CL102, CL354 and rDNA loci and the number of respective chromosomes in zigzag clover is shown in **Figure 5**.

### DNA Markers

**FIGURE 6:**
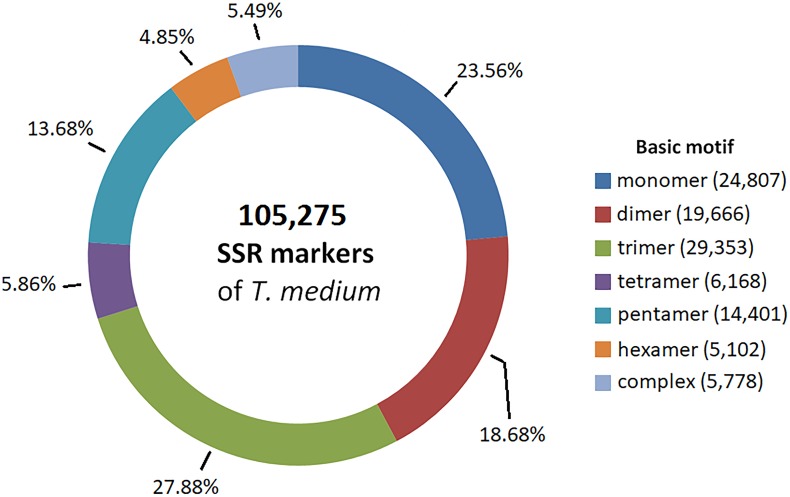
Frequency of basic SSR motifs in candidate SSR markers predicted from genomic sequences.

Partially assembled genomic sequence of 492.7 Mbp was used to predict SSR markers. We identified and designed primers for 105,275 candidate SSR markers, corresponding to 1 SSR marker every 30 kbp. The most prevalent basic motifs were trimeric, monomeric and dimeric, together comprising 70.12% of all SSR markers. A comprehensive summary of the characteristics of the predicted SSR markers is available in **Figure 6**. The predicted SSR markers are available in Supplementary Table S5.

Single nucleotide variants were identified using the coding sequence of red clover (Ištvánek et al., 2014), which enabled the identification of species-specific and interspecific candidate SNP markers in zigzag clover. A total of 1,173,317 variants were found, consisting of 133 InDels and 1,173,184 SNVs (24,592 SNVs were multiallelic). Compared to the 418 Mbp-long reference red clover genome and 3,152 Mbp-long zigzag clover genome, the predicted SNVs represent the frequency of 1 SNV every 42.3 bp and 2.7 kbp, respectively. SNVs were also differentiated to transitions and transversions based on the nature of alternative alleles. Transitions were more prevalent in zigzag clover, with the most frequent shifts being between adenine and thymine. Species-specific SNVs (707,208 SNVs; 61.57%) were also more prevalent than interspecific (441,384 SNVs; 38.43%). The mean density of species-specific SNVs in the used reference sequence was 1 SNV every 70.1 bp and 1 SNV every 112.4 bp in interspecific SNVs. The statistics of predicted SNVs in zigzag clover are shown in Supplementary Table S6. A complete list of predicted SNVs has been deposited in the Figshare depository and is available from https://figshare.com/s/c428b0ab29c37454e438.

## Discussion

In our study, the genome of zigzag clover was sequenced using a standard Illumina sequencing workflow and assembled into a partial genomic sequence of 492.7 Mbp. As a result of several conditions, such as the very large haploid size of zigzag clover genome, polyploid nature, high proportion of repetitive sequences, cross-pollination and use of a single sequencing library, final *de novo* assembly is very fragmented, does not cover the whole genomic sequence and thus is not suitable for the comprehensive annotation. However, it is sufficient for comparative purposes and characterization of repeat content that can provide us with highly valuable information about the species-specific repeats. Such repeats can be further utilized for the future precise assessment of the hybrid state of *T. pratense* × *T. medium* progeny as well as can help to understand former genomic changes that occurred during red clover and zigzag clover speciation. Although the zigzag clover genome (3,154 Mbp) is currently the largest sequenced genome in legume family, the proportion (46.74%) of fully annotated repetitive elements described in our study is comparable to that of other leguminous species (*G. max* 1.1 Gbp with 59% repetitive content (Schmutz et al., 2010), *C. cajan* 833.07 Mbp with 51.67% (Varshney et al., 2012), and *C. arietinum* 738.09 Mbp with 49.41% (Varshney et al., 2013). However, a detailed inspection was performed only for clusters containing more than 0.1% of analyzed reads, and many clusters representing repeat elements with a very small abundance were not inspected. This overall repeat content might be slightly underestimated because of the low number of reads included in the analysis (only 0.1× coverage). An analysis of higher proportion of reads was not possible due to RepeatExplorer capacity limitations. Therefore, it is likely that the genome of zigzag clover contains more repetitive elements, presumably almost 70% of the genome, as shown in **Figure 1**. The most prevalent repetitive elements in zigzag clover are Ty3/Gypsy retrotransposons (28.14%), such as in the majority of sequenced legumes (Sato et al., 2008; Schmutz et al., 2010; Young et al., 2011; Varshney et al., 2012, 2013), except for red clover, where Ty1/Copia retrotransposons are the most abundant (Ištvánek et al., 2014). On the other hand, the zigzag clover genome possesses fewer retrotransposons from the Ty1/Copia lineage (7.80%) and DNA transposons (2.89%) compared to red clover (12.22 and 6.07%, respectively) (Ištvánek et al., 2014). However, both species had mostly PIF/Harbinger transposons and CACTA the least frequently (unlike other legume species (Schmutz et al., 2010; Young et al., 2011; Varshney et al., 2012, 2013), even though their frequencies were very different. Compared to other legume species, zigzag clover had the smallest content of DNA transposons, as 16.50, 4.53, 3.40, and 3.31% DNA transposons were identified in the genomes of *G. max* (Schmutz et al., 2010), *C. cajan* (Varshney et al., 2012), *M. truncatula* (Young et al., 2011), and *L. japonicus* (Sato et al., 2008), respectively.

Repeat content characterization performed as a comparative approach (**Table 1**) showed some interesting dissimilarities between the results obtained from individual red clover (Ištvánek et al., 2014) and zigzag clover analyses. The most striking dissimilarity is a significant difference between the overall repeat content of both species. While the red clover repeat content represented 45.14% (Ištvánek et al., 2014), which was almost the same as that of zigzag clover (46.74%), clustering performed as a comparative approach showed a difference of 6.7% in terms of non-singlet reads and even 13.72% for 336 largest clusters. Another significant difference could be seen in the prevalence of individual DNA transposon lineages. While both clovers had the PIF/Harbinger transposons as the most prevalent if considered individually, in the comparative analysis, none of these species had this lineage as the most prevalent. We presume that this difference was caused mainly by the divergence of species-specific PIF/Harbinger transposons, which led to their assignment into different clusters. These clusters were then too small to be fully annotated.

A comparative analysis of both repeat contents showed that major differences between these clovers included the expansion of Ty3/Gypsy retrotransposons, specifically 6.65% in red clover and 26.29% in zigzag clover. In absolute numbers, Ty3/Gypsy spanned approximately 54 Mbp in red clover, while in octoploid zigzag clover, it was more than 766 Mbp. We presume that this dramatic difference in proportions of Ty3/Gypsy elements, especially the lineage chromovirus, is the main cause of the increased zigzag clover genome size. These results agreed with other comparisons of related species with different genome sizes, such as *Oryza sativa* and *O*. *australiensis* (Piegu et al., 2006; Zuccolo et al., 2007), *Arabidopsis thaliana* and *A*. *lyrata* (Hu et al., 2011), *Zea mays* and *Z*. *luxurians* (Tenaillon et al., 2011), and species of the *Orobanchaceae* family (Piednoël et al., 2012). The observed dominance of LTR retrotransposons in the fraction of highly repeated sequences has been previously shown to be a common feature of higher plant genomes in which retroelements represent one of the major forces driving genome size evolution (Hawkins et al., 2006; Neumann et al., 2006).

A comparative analysis of both repeat contents was used to select both red clover- and zigzag clover-specific repetitive elements. We successfully identified seven red clover-specific repetitive elements spanning 2.83% of its genome and 45 zigzag clover-specific repetitive elements spanning 10.10% of the zigzag clover genome, representing approximately 23 and 294.4 Mbp of their genomes, respectively. This higher proportion of zigzag clover-specific repeats also contributed to the increase in the genome size and probably assisted in the evolutionary diversification of both clovers (Kraaijeveld, 2010).

**FIGURE 7:**
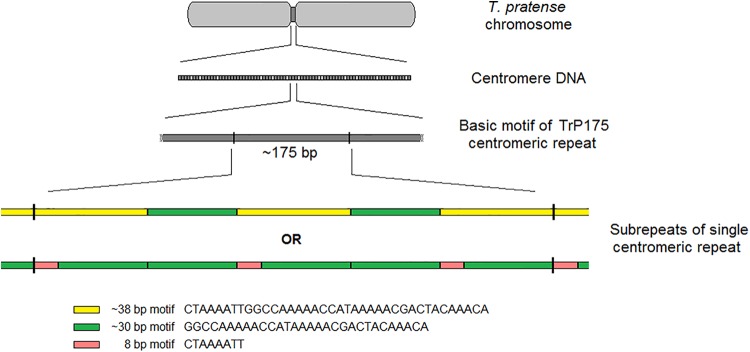
Summary of centromere-specific satellite element TrP175 derived from CL12.

The validation of selected elements was performed via FISH with fluorescent-labeled probes designed from corresponding sequencing data. FISH validation confirmed the species-specificity of all 6 and 18 elements of red clover and zigzag clover, respectively. We presumed that the CL12 repetitive element with a basic motif of 38-nt was the main repetitive element of the centromere in red clover. However, other studies have reported repetitive elements directly associated with centromere structures of different lengths, generally approximately 180 bp (Wang et al., 2009; Mehrotra and Goyal, 2014; Plohl et al., 2014), resembling the length of DNA wrapped around one nucleosome (Kubis et al., 1998; Macas et al., 2002). After the detailed reanalysis of CL12, we were able to find other basic repetitive motifs of approximately 175 bp (TrP175), consisting of three copies of our analyzed 38-nt-long element interrupted with two copies of 30-nt-long AT-rich elements. This 30-nt-long element was only a shorter version of our 38-nt-long element, lacking its first 8-nt. All 30-nt-long copies were almost identical, with only minor shifts in the position of GC bases within poly-AT tracts or prolongation in individual poly-AT tracts. The resulting structure of centromere-specific satellite repeat TrP175 derived from CL12 is thus summarized in **Figure 7**. Centromeric repeat TrP175 resembled centromere repeat of another clover species, TrR350, which was identified in *T*. *repens* (Ansari et al., 2004). They were similar in terms of GC content (32% in TrR350 and 33% in TrP175), inner structure comprising shorter submotives (24-nt long in TrR350) and high occurrence of tracts similar to the CAAAA motif. TrR350 was present only in the *Trifoliastrum* section [according to Ellison et al. (2006) taxonomy]; newly annotated TrP175 could play the same role in other *Trifolium* sections, which will be further inspected in the future.

Repeats derived from CL9 (TrM378) and CL17 (TrM300) showed a very specific localization into centromeres of zigzag clover chromosomes. Eight chromosomes exhibited a higher proportion of TrM300, while the remaining 24 chromosomes exhibited a higher proportion of TrM378. These results are rather rare, as most plant centromeres tend to homogenize their basic tandem repeat motifs. Similar results were discovered in potato, in which six different centromeres possessed at least three different centromere-specific tandem repeats (Gong et al., 2012; Wang et al., 2014). TrM300 and TrM378 elements were also present only on half of all chromosomes, suggesting that the other half of all chromosomes could have a different origin and thus a different type of centromeric repeat. This would mean that zigzag clover comes from the hybridization of two different species and is thus an allopolyploid and that the naturally occurring homogenization of the centromeric repeat motif is somehow prevented. Another explanation could be the considerable divergence of the original centromeric repeat, in which some centromeres of a single species had a different basic repeat motif than that of others, as previously reported (Macas et al., 2010). This other tandem repeat was not zigzag clover-specific and could also be present in red clover, meaning that it was not selected for validation in the first place. Another hypothesis is that half of chromosome centromeres without TrM300 and TrM378 lack a tandem repeat at all, and these centromeres are almost exclusively composed of single- or low-copy sequences, which were previously discovered in potato (Gong et al., 2012; Wang et al., 2014). All four newly discovered tandem repeats, TrM300, TrM378, TrM179 and TrM60 (**Table 4**), as well as previously reported 5S and 45S rDNA were used for the closer inspection of zigzag clover chromosomes. A total of 12 chromosomes were left without any hybridization signal; the remaining 52 chromosomes carried one or a combination of two tested elements. Using all of these various repeats, we were able to separate highly uniform 64 chromosomes into eight types of chromosomes (**Figure 5**). Even though this method cannot distinguish all of the individual chromosomes, the results imply that the zigzag clover genome underwent substantial chromosome rearrangements and cannot be counted as a true octoploid because such a complex karyotype cannot be reduced to a haploid set of eight chromosomes.

DNA markers have a broad spectrum of use in both research and practice. They are used for QTL mapping (Řepková et al., 2006a; Soldánová et al., 2013; Zhao et al., 2013), the deduction of evolution relationships (Isobe et al., 2012), variability assessment and genotypization of primary breeding material (Younas et al., 2012; Cidade et al., 2013), marker-assisted selection in breeding generations and even gene pyramiding (Qi et al., 2015). Based on NGS technology, the number of newly discovered DNA markers substantially increased (Zalapa et al., 2012). In zigzag clover, partially assembled genomic sequence was used to predict SSR markers. The high frequency of predicted SSR markers (1 SSR marker every 30 kbp) can be successfully utilized in breeding programs. Candidate SNVs can be used for the additional saturation of zigzag clover genome by SNPs using high-throughput screening technologies, e.g., SNP arrays (Víquez-Zamora et al., 2013; Yu et al., 2014). The classification into species-specific and interspecific categories also enables the study of differences between clover species and their use in breeding programs encompassing an available interspecific hybrid of red and zigzag clover (Řepková et al., 2006b; Jakešová et al., 2011). However, the number of predicted SNVs is influenced by many circumstances, such as the number of individual plants analyzed, natural sequence variability in the population and allogamy. Compared with other plant species [*Prosopis alba*: 1 SNP every 2,512 bp (Torales et al., 2013); *Capsicum annuum*: 1 SNP every 2,253 bp (Ashrafi et al., 2012); oak: 1 SNP every 471 bp (Ueno et al., 2010); and *Eucalyptus grandis*: 1 SNP every 192 bp (Novaes et al., 2008)], SNV density found in zigzag clover (1 SNV every 70.1 bp) was the highest; however, only one clone was analyzed without establishing frequency of occurrence. The polyploid nature and lack of artificial selection in zigzag clover may also be the reason. On the other hand, great sequence variability was discovered also in red clover (1 SNP every 144.6 bp (Ištvánek et al., 2017). The high density of SNP markers provides us with an opportunity to study specific genes, key enzymes and even whole biosynthetic and metabolic pathways.

## Author Contributions

JD prepared biological material, performed repeat content characterizations and comparative analyses and designed and performed FISH experiments. JI processed raw sequencing data, assembled the partial genomic sequence, and identified DNA markers. JŘ and JN designed the study and supervised all aspects of the presented analyses. All of the authors contributed to the analysis of data and the writing of the manuscript and approved the final manuscript.

## Conflict of Interest Statement

JN was employed by company Agricultural Research, Ltd., Troubsko, Czechia. The other authors declare that the research was conducted in the absence of any commercial or financial relationships that could be construed as a potential conflict of interest. The reviewer AG and handling Editor declared their shared affiliation.

## Footnotes

1 http://www.bioinformatics.babraham.ac.uk/projects/fastqc/

2 http://hannonlab.cshl.edu/fastx_toolkit/

3 https://galaxyproject.org/

4 ftp://occams.dfci.harvard.edu/pub/bio/tgi/software/

5 http://broadinstitute.github.io/picard/
