# Supplementary material for: Red Clover (Trifolium pratense) and Zigzag Clover (T. medium) – A Picture of Genomic Similarities and Differences
Source: Front Plant Sci. 2018 Jun 5;9:724. doi: 10.3389/fpls.2018.00724 (PMC5996420; doi:10.3389/fpls.2018.00724)
Supplement: Supplementary file 1 [file Table_1.DOCX]

**TABLE S1** Zigzag clover genome assembly features.

| **Assembly features** | **Contigs ≥ 200 bp** | **All contigs** |
| --- | --- | --- |
| Number of scaffolds | 1,354,235 | 33,648,187 |
| Total span (Mbp) | 492.7 Mbp | 3,619 Mbp |
| Average scaffold length (bp) | 368 bp | 107.6 bp |
| N50 (scaffolds) (bp) | 319 bp | 121 bp |
| N90 (scaffolds) (bp) | 211 bp | 65 bp |
| Longest scaffold (bp) | 37,772 bp | 37,772 bp |
| Number of contigs | 1,471,620 | 33,802,156 |
| Average contig length (bp) | 334.8 bp | 107 bp |
| N50 (contigs) (bp) | 300 bp | 121 bp |
| N90 (contigs) (bp) | 211 bp | 65 bp |
| Longest contig (bp) | 15,617 bp | 15,617 bp |
| GC content (%) | 33.4% | 35.9% |
