# Supplementary material for: Red Clover (Trifolium pratense) and Zigzag Clover (T. medium) – A Picture of Genomic Similarities and Differences
Source: Front Plant Sci. 2018 Jun 5;9:724. doi: 10.3389/fpls.2018.00724 (PMC5996420; doi:10.3389/fpls.2018.00724)
Supplement: Supplementary file 2 [file Table_2.DOCX]

**TABLE S2** Repeat composition of the zigzag clover genome estimated from the Illumina sequencing data.

| Classification | | |  | Genome proportion (%) |
| --- | --- | --- | --- | --- |
| Repeat type | Family | Lineage |  | *T*. *medium* |
| Retroelements |  |  |  | **36.66** |
|  | Ty3/Gypsy |  |  | **28.14** |
|  |  | Chromovirus |  | 17.8 |
|  |  | Tat/Ogre |  | 6.12 |
|  |  | Athila |  | 4.14 |
|  |  | other |  | 0.08 |
|  | Ty1/Copia |  |  | **7.8** |
|  |  | Maximus/SIRE | | 5.24 |
|  |  | Angela |  | 0.83 |
|  |  | Bianca |  | 0.82 |
|  |  | Tork |  | 0.36 |
|  |  | Ivana/Oryco |  | 0.16 |
|  |  | AleII |  | 0.15 |
|  |  | TAR |  | 0.08 |
|  |  | AleI/Retrofit |  | 0.03 |
|  |  | other |  | 0.13 |
|  | LINE |  |  | 0.51 |
|  | SINE |  |  | 0 |
|  | other |  |  | 0.21 |
| DNA transposons | |  |  | **2.89** |
|  | PIF/Harbinger |  |  | 1.04 |
|  | Mutator |  |  | 0.62 |
|  | RC/Helitron |  |  | 0.5 |
|  | hAT |  |  | 0.18 |
|  | Mariner |  |  | 0.11 |
|  | CACTA |  |  | 0.06 |
|  | other |  |  | 0.38 |
| Satellite repeats |  |  |  | 0.73 |
| rDNA |  |  |  | 1.67 |
| Unclassified |  |  |  | 4.79 |
| **Total** |  |  |  | **46.74** |
