# Supplementary material for: Red Clover (Trifolium pratense) and Zigzag Clover (T. medium) – A Picture of Genomic Similarities and Differences
Source: Front Plant Sci. 2018 Jun 5;9:724. doi: 10.3389/fpls.2018.00724 (PMC5996420; doi:10.3389/fpls.2018.00724)
Supplement: Supplementary file 3 [file Table_3.DOCX]

| Element | Cluster | Specifity | Monomer length (bp) | Monomer sequence |
| --- | --- | --- | --- | --- |
| TrP175 | CL12 | *T.* *pratense* | 38 | CTAAAATTGGCCAAAAACCATAAAAACGACTACAAACA |
| TrM179 | CL102 | *T.* *medium* | 179 | CCGCTCTACGTATTTCGGTAGTGCCCTACAAAAAAAATGGAGCATAAATTCTATTCCTATGCGCCGCGGTAGCCAAGTTTCAGTTTCTCGACCTAGCCGCGACGTTGAAAACGCGTTCGTCGGGTAAAAACAATGAAAACTTGCAACGGTAAGTCGGAATGCTACGAAATTTTGTAGAC |
| TrP1586 | CL167 | *T.* *pratense* | 1586 | CCCTGATCTCCTTCAAAGCCCTTCATATTCTCAGCAACCTTGGAAGTTCTCGGCTTGACGAAGAACTTGTTGCTCTGAATGTAGCCATTGGTACCAGACCCTCTTGGAGTTTGTAACCCTATTCCGTTATACATATTGTGACAAAGCAACAACTGCAATTGAAAACCCAAAAAATCATATAAGAAACCCTAATATCAACTTAAATTCACAGATCTTAAATAAATAAATATATATCTGATGCAATTAACAAATTGAACGGCAAAATGAAAAAAGATGAGAAACCCTAATTTAAACGACGGCAATTAACGGATAAAAAGCTGAGCGTAGAAGAAAAAGAAGAATATGTCTGAATCTGTGATCCGAGACAAGAAAACGAAGAGTAGTAATACCTGTTATCGATGTGGAAGAAGAATTATCGCGACTCAAAGAAATAGAGAAGCCACAATGTTGTTGATACTTTATTTATGATGATGATGATATTTGATTTGATATTTTATCATATACTTATGGCTTAATTAGTTAAATGGTCCCTTAGAGTGTGTTTGGATGAGGATTTTATCATATACTTAGGTTATTTTGTCGATTTTAGCATCCAAACGCACTGTAAGGGACCATTTAATGATGCTTAATCACTTTAATTCGATTCGCACCGTGTTATTCAATTAGTTTATCGCTTGCATAGATTACATTGAATAGGAATTGACATAAACTTTGAGCGCTTAGCAAAAAGTGTTGATTGAATTCAGTAAGGATATTATTCTGTCATTAGTAGCGCTTGTTTACTATTGCCTATGATTAGTTGAACGCATTTGACGCTTGTTTGAATGGAATTCGTATAAAATTGGTAACGCTTGTTTAATTCGGTTTTATAAATCAACCAGAACATAAGCATATAACGAATTTATTCTTTGTGAACCATATGATAGAATCAAGAACGAGTTACCTAAGCCAATGAATTGATCGTTTTAAATCAATTAAATTTCACATTTTTCTTACGTTCTGTTAACAAACCAAACCCCCCCATAATTTACAGTTTTTGAATTGTTTCTAATATAGATAAGCCGATTGTGAGTCTTCGGAGACGACCAAGGTTAACTGCCTTGTATTACTTTTTATTTAAATTATTATTTGACCACGAAACGACCGTGATCACATACTATTCAACTTTAATTAATAATTATAATCATATATATAATCTCCTACAACAACAATAACACCAATAAAAATAATTTCTCCATAATCAAAAACAAAAAGCTATGCACAACAAAAACCAATTAACAATTAATACACTTCAAAAATTTTGGTAAAGCAATGACAGAACCAACAAATTCATCAATTGGAATCATATGTCATACTCATACACAAATATCTAAAATCCAAAAACTCACTTGTCCAAATTAGAATCATTTTCGTCAGCTGCAGCCTCCAAGCTATTGCGAGCCTCCTCGAGTTTCTCAGCGATCTCAGCATCAGTATAACCCTGATCAATTAACTTGTCTTCAAGAATAACAAGTTTGAGCTGAATCTGACGCTTACGATCATGCTCAAGAATCTCTTTGTTAGCCTTTCTGGAAACACCAGCAGTA |
| TrP29 | CL198 | *T.* *pratense* | 29 | TCCCTAAATTGAGCTTATGTCCCTCGGTG |
| TrM60 | CL354 | *T.* *medium* | 60 | TTTTATCCATCATTAGCATCTTCAGGCTCTGCCCTAACCTATAGAGGTCTTCCTTTCACC |

**TABLE S3** Consensus monomer sequences of species-specific tandem repeats.
