# Supplementary material for: Red Clover (Trifolium pratense) and Zigzag Clover (T. medium) – A Picture of Genomic Similarities and Differences
Source: Front Plant Sci. 2018 Jun 5;9:724. doi: 10.3389/fpls.2018.00724 (PMC5996420; doi:10.3389/fpls.2018.00724)
Supplement: Supplementary file 4 [file Table_4.DOCX]

| Cluster | Forward primer | Reverse primer | PCR product length (bp) |
| --- | --- | --- | --- |
| CL9 | ACTTTTGATCTGGTTATCTCT | ACTGTATATGAATCGAGAAGCA | 378 |
| CL17 | CTGTTAGTAAGCTATTAGAAGT | ATTTAACTTATCTGCACTATCTT | 300 |
| CL50 | TCGATAGGCTCCTCCTTCAAAAG | GTGACAAGTGCCAACGATTTTCA | 851 |
| CL53 | GGTTGGTTGTTTCTTCTGGTTCT | CTTTGCACTCTTCACCAGAAACT | 1,026 |
| CL55 | GTCTTCGGCAGTATGAATCTAGC | ATCTTAACCCGCTTAAAAGACCG | 1,098 |
| CL64 | AATGTACGCCACGATGGTTTATG | GCCAAACCACCACTTTCTACAC | 923 |
| CL102 | CTCTACGTATTTCGGTAGTGCCC | TCATTGTTTTTACCCGACGAACG | 132 |
| CL106 | TGTGTCTGATTGGATAAAGGGT | AAAGAAAAGCTCTGACAAAAGCC | 1,052 |
| CL110 | CAATGTTTTACTTGATTTGCTGTGA | CAAAGAAAACAAGTGGAGAAAGTGA | 754 |
| CL122 | TACTACGACCCCAAAACCTGAAG | TGTTCCATTACCTTTCGAGAATGC | 957 |
| CL127 | ACAAGTGCATAGAATCCTAGGGT | CCATCTTCTGAACCTCAACCAAA | 940 |
| CL140 | TTCCTCTAAAATCCCCCTCCTTG | CTGGAGGACCAATAGAAAGGGAA | 807 |
| CL146 | CTTCTGCTTCAACGGTAACTCTG | CAAGAGAATGGAAGCCGCAATAA | 1,109 |
| CL150 | TCCTTGGGGTTTGAAGTTTGTTG | CTCACCTTTTTACCCTCGATTCA | 992 |
| CL153 | TCACAAACTCTTCAACATCCTGTC | TCACTGTCTGGATTCTCTTGGGA | 727 |
| CL164 | GTACTCCGACCAATTACCAAGCA | GGAAAGGGCAAGAAAACCAACTC | 750 |
| CL167 | CAACCTTGGAAGTTCTCGGC | CGGTCGTTTCGTGGTCAAAT | 1,112 |
| CL172 | CAAGCACACTCTTCTCCGTTTAT | GGATTTGGATAATGACAGGGCAA | 671 |
| CL195 | ACCCTACTACTGATCCTGTCCAA | GAAAAATCAACACAGCAGCCTCT | 960 |
| CL196 | TGTATTTGCAGAGGAAGCAGAAT | AAATAACAAAAGTGCAACCATTTCA | 811 |
| CL197 | TGACACTGAGGTTGTAGATGTTGA | TCTGGTTTGCAGTGATTGTCTTG | 800 |

**Table S4 Cluster-specific primers used for the amplification of DNA for FISH probes.**
