# Supplementary material for: Red Clover (Trifolium pratense) and Zigzag Clover (T. medium) – A Picture of Genomic Similarities and Differences
Source: Front Plant Sci. 2018 Jun 5;9:724. doi: 10.3389/fpls.2018.00724 (PMC5996420; doi:10.3389/fpls.2018.00724)
Supplement: Supplementary file 6 [file Table_6.DOCX]

**TABLE S6** Statistics of predicted single nucleotide variants (SNV) in zigzag clover using the red clover coding sequence as a reference.

| Transition | | | Transversion | | |
| --- | --- | --- | --- | --- | --- |
| SNV | Number | Frequency (%) | SNV | Number | Frequency (%) |
| A↔G | 365,267 | 50.08 | A↔C | 114,114 | 27.22 |
| species-specific | 232,933 | 63.77 | species-specific | 67,074 | 58.78 |
| interspecific | 132,334 | 36.23 | interspecific | 47,040 | 41.22 |
| C↔T | 364,113 | 49.92 | C↔G | 72,799 | 17.37 |
| species-specific | 229,087 | 62.92 | species-specific | 39,968 | 54.90 |
| interspecific | 135,026 | 37.08 | interspecific | 32,831 | 45.10 |
| **Total** | **729,380** | **62.16** | G↔T | 96,357 | 22.99 |
|  |  |  | species-specific | 56,756 | 58.90 |
|  |  |  | interspecific | 39,601 | 41.10 |
| multiallelic SNVs: | 24,592 | 2.10 | T↔A | 135,942 | 32.43 |
| InDel number: | 133 | 0.01 | species-specific | 81,390 | 59.87 |
|  |  |  | interspecific | 54,552 | 40.13 |
|  |  |  | **Total** | **419,212** | **35.73** |
