# Supplementary material for: Red Clover (Trifolium pratense) and Zigzag Clover (T. medium) – A Picture of Genomic Similarities and Differences
Source: Front Plant Sci. 2018 Jun 5;9:724. doi: 10.3389/fpls.2018.00724 (PMC5996420; doi:10.3389/fpls.2018.00724)

FIGURE S1 PCR validation of species-specificity and predicted length of individual *T. medium*- and *T. pratense*-specific elements. **a** Validation of *T. medium*-specific elements. **b** Validation of *T. pratense*-specific elements. **M** *T. medium*, **P** *T. pratense*.

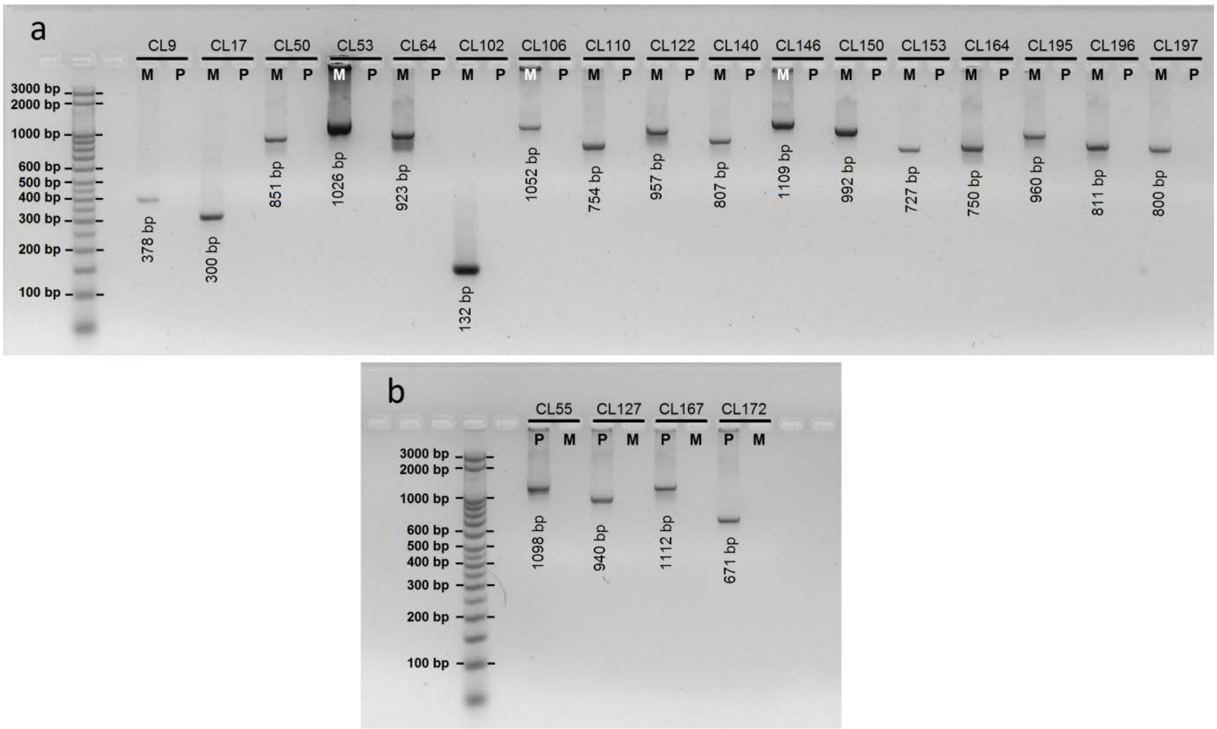

Supplement: Supplementary file 7 [file Image_1.PDF]
